# Study on the influence of assistant experience on the quality of colonoscopy: A pilot single-center study

**DOI:** 10.1097/MD.0000000000017747

**Published:** 2019-11-11

**Authors:** Lixia Fu, Mugen Dai, Junwei Liu, Hua Shi, Jundi Pan, Yanmei Lan, Miaoxia Shen, Xiaoduo Shao, Bin Ye

**Affiliations:** a Department of Gastroenterology; b Department of Nursing, The Fifth Affiliated Hospital of Wenzhou Medical University, Lishui, Zhejiang, China.

**Keywords:** assistant, colonoscopy, experience, quality

## Abstract

**Background and objective:**

Colonoscopy is the most important method for the diagnosis and treatment of intestinal diseases, and there are many factors affecting the quality of examination. Although the assistant is one of the factors influencing the quality of colonoscopy, there are few studies on the effect of different assistants with different experiences on the quality of colonoscopy. Therefore, the study was aimed to research the correlation between different assistants with different experiences and the quality of water-injection colonoscopy.

**Method:**

In this study, a single-center randomized controlled trial was conducted to analyze the key quality indicators (the rate to arrive cecum, time to arrive cecum, total operation time, detection rate of polyps, detection rate of adenoma, pain score, operation satisfaction, and the pressure on abdomen) of patients who underwent water-injection colonoscopy under non-sedation from January 2018 to June 2018 in the center. Patients were randomly assigned to different assistant groups based on the actual working period of 6 months (0∼6 months inexperienced assistant group and assistant group with more than 6 months of experience). Through fitting the bivariate and multivariate logistic regression models, the differences between the two groups and the effects on the key quality indicators of colon examination were analyzed.

**Results:**

A total of 331 patients who were eligible for non-sedation colonoscopy were randomly assigned to the experienced assistant group (n = 179) and the inexperienced assistant group (n = 152). Among them, 103 cases of polyp and 70 cases of adenoma were detected. The rate to arrive cecum, polyp detection rate and adenoma detection rate were compared between the two groups during operation (*P* > 0.05). However, there were significant differences in the time to arrive cecum, patients’ satisfaction with operation, pain score and abdominal pressure (*P* < .05). In the inexperienced assistant group, 20% of the operation time was one standard deviation higher than the mean value, while the experienced assistant group was 12% (339 s vs 405s, OR 0.541, 95% 0.295–0.990). Compared with the inexperienced assistant group, patients in the experienced assistant group had higher operational satisfaction (98.32% vs 92.11%, OR 0.199, 95% 0.055–0.718) and lower pain score (0.3 vs 0.49, OR 1.993, 95% 1.52–3.775). All relations remained unchanged after adjusting for potential confounders.

**Conclusion:**

The assistant is a key factor in the quality of colonoscopy, especially in the case of non-sedating colonoscopy. The experience of the assistant is related to the time to arrive cecum, the degree of pain and the overall satisfaction of patient with the operation. The assistant should be subject to the quality supervision of the endoscopic inspector. Proof of human Clinical Trial Registration: The institutional review board of Fifth Affiliated Hospital of Wenzhou Medical College, Zhejiang Province, China approved the study. The study is registered on. Chinese Clinical Trial Registry (ChiCTR1800015650).

## 1 Introduction

Colonoscopy is an economic and effective method for the prevention and screening of colorectal cancer.^[1]^ Therefore, it is recommended that healthy people over 40 years old and those with symptoms should undergo colonoscopy.^[2]^ Meanwhile, high-quality colonoscopy and good colonoscopy experience can effectively find the intestinal lesions and improve the follow-up rate of patients.^[3,4]^ Therefore, more and more attention has been paid to the quality of colonoscopy in recent years. On clinic, the quality of colonoscopy can be improved by training endoscopists.^[5]^ Colonoscopy assistants and endoscopists complement each in their work and play an important role as: educate the patients with colonoscopy; assist patients with posture changes; press abdomen if necessary; placate patients during surgery; intravenous intubation; give medicine to calm down, etc.^[6]^ However, there are few domestic studies on whether the experience of colonoscopy assistants affects the quality of colonoscopy. The study was aimed to determine whether the experience level of the assistant for colonoscopy was related to the quality of screening colonoscopy. The indicators include pain score, surgery time (time to arrive cecum and total operation time), rate to arrive cecum, and operational satisfaction. Previous studies have shown that the quality of colonoscopy is improved when experienced assistants assist endoscopists, and these studies combined the two into studies do not represent the influence of assistant experience on the quality of colonoscopy. The study was aimed to determine whether the experience of the assistant was related to the quality of the colonoscopy examination after removing all confounders such as the colonoscopy surgeon.

## 2 Method

### 2.1 Conditions of patients

**Figure 1:**
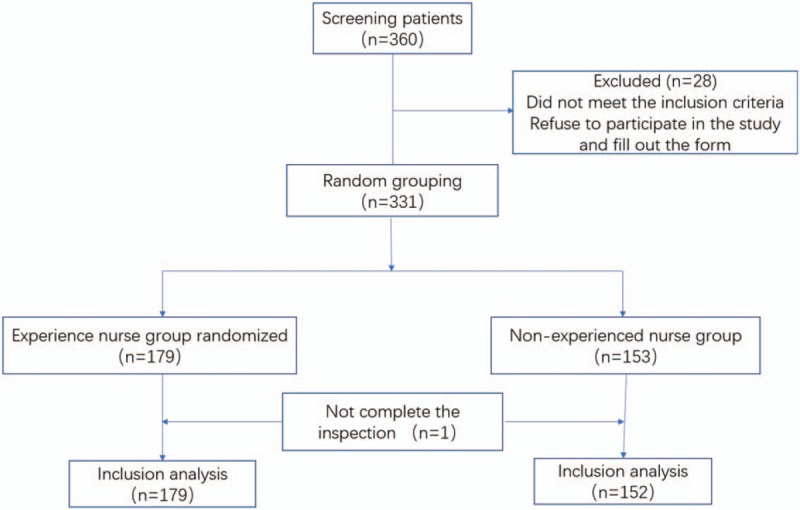
Study flow diagram.

Patients who underwent non-sedating colonoscopy at the center from January 2018 to June 2018 were selected, aged 18 to 85 years (Fig. 1). Exclusion criteria: Patients with painless colonoscopy; Boston Bowel preparation (Boston Bowel Prep < 2 points, for any part); Refuse to sign the informed consent; Familial polyposis; Inflammatory bowel disease; Active gastrointestinal bleeding; Pregnancy; Previous colon or rectal resection; Systemic or psychiatric disorders.

### 2.2 Randomization and manipulation

According to the random list generated by the computer, patients were randomized (1:1) into the group of inexperienced assistants with less than 6 months of experience and the group of experienced assistants with more than 6 months of experience. For the purpose of the study, we limited the group of assistant experience to 6 months, the group of inexperienced assistants with less than 6 months of experience and the group of experienced assistants with more than 6 months of experience. Each group was fixed with two assistants because six months is a threshold level for many performance parameters. The operation was performed by a certified endoscopic physician, with more than 10,000 cases of experience in colonoscopy operation. The assistant was randomly assigned to the physician. Before the operation, the assistant recorded the questionnaire which involved that surgery history of patients, the purpose of colonoscopy, complications and current medications. Colonoscopy began in the left lateral decubitus position without preoperative medication. The colonoscopy was produced by Olympus company (PCF-Q260AZI, PCF-H290I, GIF-Q260J). Water injection began in the rectum (37°C of water temperature), and the water pump was closed after that the water had passed the sigmoid colon, and the water injection was changed into gas injection. According to the situation of admission, if the admission is difficult, the assistant carried out the auxiliary pressure. Abdominal press and postural translocation were performed in both groups. Operation time was expressed as seconds, and the time to arrive cecum was defined the time from rectum to cecum. Colonoscopy retracting time means time to start retracting. Polyps and adenomas were counted during the retracting process.

### 2.3 Operational satisfaction

After the completion of colonoscopy, the operational satisfaction was assessed by the score (≥2 means satisfied, <2 means dissatisfied): 0 means extremely dissatisfied; 1 means generally dissatisfied; 2 means generally satisfied; 3 means very satisfied.

### 2.4 Pain assessment and sedation

Pain was assessed using a numerical pain score (NRS) (0 point: no pain; 1 to 3 points: mild pain (pain does not affect sleep); 4 to 6 points: moderate pain; 7 to 9 points: pain (cannot fall asleep or wake up during sleep); 10 points: severe pain). Before the operation, the assistant explained the NRS scoring method to the patients and guided them to express their actual feelings of pain. The assistant made scores through looking directly at the patients’ face and asking questions. When the NRS score is greater than or equal to 6 points, a tranquilizer such as propofol can be provided intravenously. After the operation, the assistant recorded the patients’ overall scores for subsequent analysis.

### 2.5 Endpoint of study

The main endpoints were time of admission, abdominal press, pain score, rate to arrive cecum, and operational satisfaction.

Secondary results were polyp detection rate, adenoma detection rate, retracting time, and sedation.

Other covariates of interest included the quality of bowel preparation assessed by operator, age, gender, and surgical history of patients.

### 2.6 Statistical analysis

SPSS23.0 was applied for statistical analysis, and *P* < .05 indicated statistical significance. Mean plus or minus standard deviation was used for normal distribution data and median for non-normal data. The comparison of the two groups of measurement data that follow normal distribution and homogeneity of variance is performed by *t* test, otherwise, the non-parametric Mann-Whitney *U* test was used. Counting data was expressed as rate, and the comparison between the two groups was performed by the four-grid table of χ^2^ test or multi-factor χ^2^ test, while N < 40 was performed by Fisher exact probability method. *P* < .05 was considered as the difference, and the pain value was calculated by mean value and 95% confidence interval. The logistic regression statistical method was used for multivariate analysis. Specially, all confounding factors were included into the initial model, and variables with OR were removed through backward elimination method. Finally, all interactions between exposed and potentially confounding covariates were evaluated through the ratio test. Because there was no obvious interaction during the operation, there was no interaction method used in the model.

## 3 Results

### 3.1 General characteristics of patients

A total of 331 patients were included in this study, and were randomly assigned to the experienced assistant group (n = 179) and the inexperienced assistant group (n = 152). The mean age of the patients was 53.44 ± 11.193. 54% were female and 46% were male. There was no statistical difference between the two groups in age. 84.9% of them had excellent or good bowel preparation (*P* > .05, OR 0.978, 95%CI 0.547–1.748), and there was no statistical difference between the two groups. 21% had abdominal surgery (*P* > .05, OR 0.904, 95%CI 0.535–1.530), and there was no statistical difference between the two groups. For the details, please see Table 1.

**Table 1:**
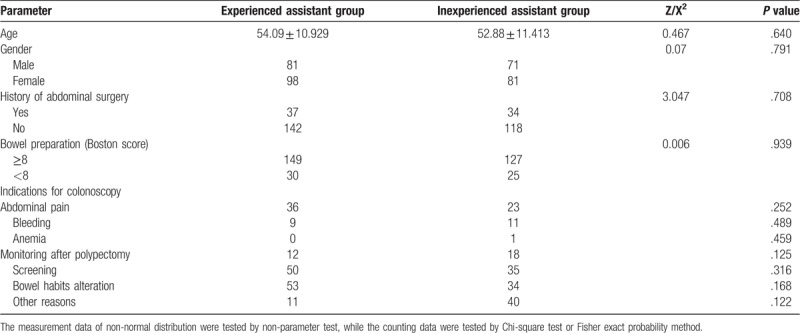
Baseline characteristics and indications of 331 patients.

### 3.2 Screening characteristics of colonoscopy

The rate to arrive cecum of colonoscopy was 100% in both groups. The average time to arrive cecum of the 2 groups was 369.92 seconds (with a range of 130∼1465 seconds), and there was a significant difference between the two groups (*P* < .01). In the inexperienced assistant group, 20% (30/152) had a standard deviation higher than the mean value, while the experienced assistant group was 12% (21/179). There was no difference between the two groups. A total of 103 cases of polyps (31%) and 70 cases of adenomas (21%) were detected during the operation, and multiple polyps or adenomas were found in 19% of the patients. The number of polyp patients in experienced assistant group was higher than that in inexperienced assistant group, and the difference was not statistically significant (*P* > .05, OR 1.207, 95%CI 0.755, 1.929). The number of adenoma patients in the experienced assistant group was higher than that in inexperienced assistant group without statistical significance (*P* > .05, OR 1.087, 95%CI 0.643, 1.849). The pain score of inexperienced assistant group was higher than that of the experienced assistant group (*P* < .05, OR 1.993, 95% 1.52–3.775) with statistical significance. Compared with the inexperienced assistant group, patients in the experienced assistant group had higher satisfaction with the operation (*P* < .05, OR 0.199, 95% 0.055–0.718) with statistical significance, as shown in Table 2.

**Table 2:**
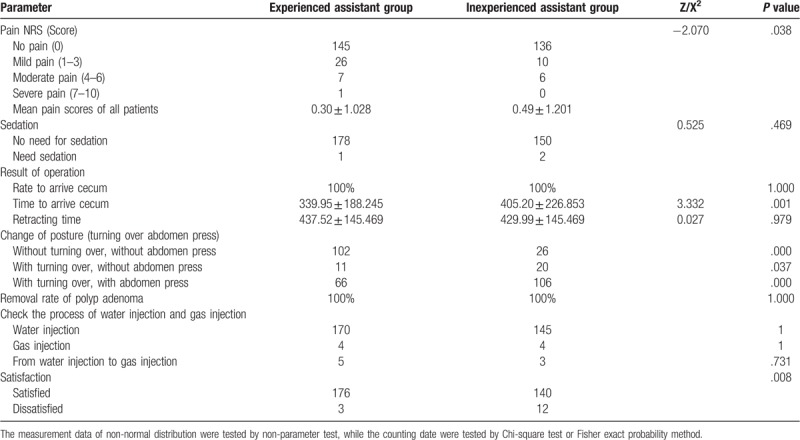
Primary outcomes, procedural outcomes.

### 3.3 Multi-analysis

The risk factors were identified by univariate analysis and multiple regression analysis was performed. The polyp detection rate and adenoma detection rate were included in the analysis regardless of the significance or not. The dependent variable was the experience of assistant (>6 months = 2, < 6 months = 1), and the gender (male = 2, female = 1), age, polyp (yes = 2, no = 1), adenoma (yes = 2, no = 1), time to arrive cecum, bowel preparation (Boston score ≥ 8 = 2, Boston score < 8 = 1), surgical history (yes = 2, no = 1), and retracting time were covariate to perform the binary logistic regression. The probability of covariate introduction and deletion was set to 0.05 and 0.1, and the backward elimination method was used to remove variables with OR. The dissatisfaction with the overall operation was related to the insufficient experience of assistant, which was not significantly changed by multivariate regression analysis, Compared with the experienced assistant group, the inexperienced assistant group had longer time to arrive cecum (OR = 1.210, 95% = 1.01–1.471), lower operational satisfaction (OR = 5.498, 95% = 1.503–20.107). Therefore, the lack of experience is a risk factor of time to arrive cecum and operational satisfaction. The model was adjusted for the age, gender, bowel preparation quality and surgical history of patients, and the OR values were basically the same, which indicated that there were few confounding factors in the model. For the details, please see Table 3.

**Table 3:**
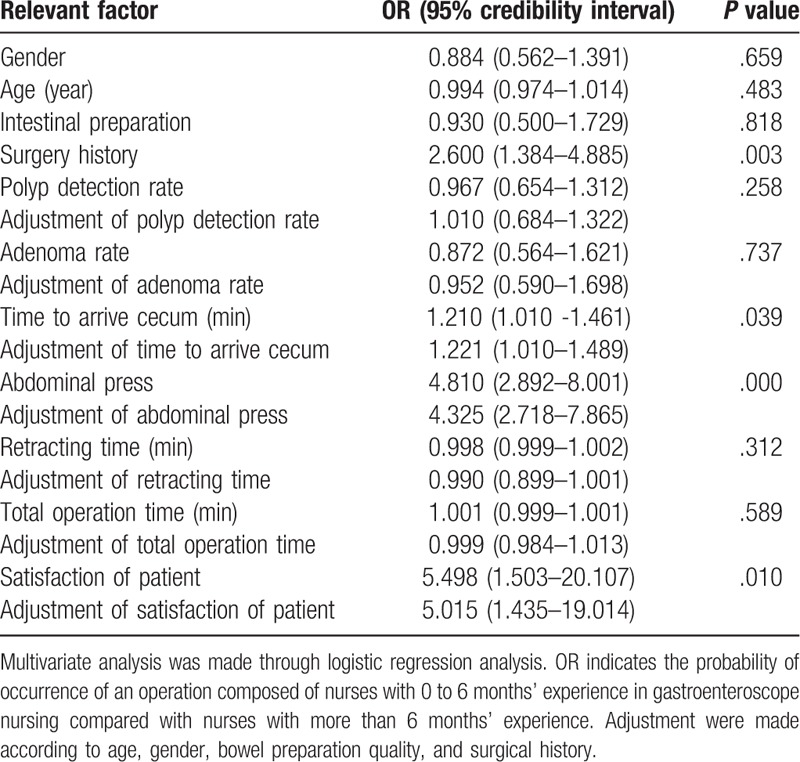
Logistic regression analysis.

## 4 Discussion

Colorectal cancer is one of the most common malignant tumors in clinic, and its incidence is increasing year by year. Reducing the incidence and mortality of colon cancer is a major clinical problem without delay.^[1,7]^ Based on the popularity of colorectal screening, endoscopic diagnosis and treatment of early colorectal cancer is an effective approach.^[8]^ No matter what screening strategy is adopted, colonoscopy and subsequent endoscopic resection of lesions are the most critical links.^[9]^ In the early 2000s, the United States began to evaluate the quality of colonoscopy, and gradually built a colonoscopy quality control system centered on the detection rate of adenomas, including the rate of cecum insertion and the time of withdrawal, etc. The system promoted the overall improvement of colonoscopy quality in the United States and ensured the effectiveness of colon cancer screening program.^[10]^ Successful colonoscopy to cecum is the basis of a complete colonoscopy, and the rate to arrive cecum is to control the quality of colonoscopy from the point of view of the completion of colonoscopy.^[11]^ There are many factors influencing the incidence of blindness. Because the colonoscopy in Europe and the United States is basically operated under anesthesia, there is a lack of data and literature on non-anesthetic colonoscopy assistants to assist abdominal compression. For the non-anesthetic water-filled colonoscopy, the clinical trials were compared with the assistants who had rich experience in abdominal assisted compression and the two groups of colonoscopy quality statistics, which were rich in experience, and there were significant differences, especially the blindness rate, the time of the lens and patient comfort. At the same time, non-anesthetic colonoscopy requires more assistants and requires more experienced assistants. As the second person in the operation, the close cooperation between the operator and the interaction between the patient and the quality of colonoscopy examination has not been reported in China. The relationship between assistant experience and the quality of colonoscopy was assessed using a single-center prospective randomized study.

In assisting a colonoscope surgeon to perform the procedure, the assistant must master the monitoring and recording of the patient's vital signs, and make the patient sedation, and assess the sedation level, and be familiar with each step of procedure.^[12]^ As the inexperienced assistant cannot master the entire operation process skillfully, it will increase the time of admission and reduce the success rate of cecal intubation. Moreover, inexperienced assistants may not be able to master the timing and location of abdominal pressure, resulting in difficulty in maintain axial shortening through the sigmoid colon increasing the difficulty of admission and resulting in the patient's adverse colonoscopy polypectomy, it will increase the time required for polypectomy.^[13,14]^ As found in this study, in the process of colonoscopy performed by experienced endoscopists, compared with the operation composed of inexperienced assistants and the operation with experienced assistants, the time and position change (turning over and pressing the abdomen) of the endoscopy increased. Furthermore, this effect persisted after controlling for known possible influencing factors.^[15,16]^ Appropriate abdominal pressure and position changes can improve the rate of cecal admission, and reduce the pain score of patient, and improve the patient's satisfaction with colonoscopy, and improve the detection of tiny polyps. The inexperienced assistant can improve the rate of blindness by increasing the time of turning over and pressing, while the time to arrive cecum is extended at the same time. The study also found that the pain score of patients in the experienced assistant group had reduced.^[17,18]^ In order to ensure the quality of non-anesthesia colonoscopy operation, the training of assistants is very important. At present, the endoscopy center lacks a formal training system for assistants. The experienced assistants teach the assistants with poor experience and carry out formal training, so that the assistants can master the regular pressing techniques and guarantee the quality of the colonoscopy operation.

There was no difference in the detection rate of polyps and adenomas between the two groups, which might be related to the direct correlation between the detection of polyps and adenomas and the experience of endoscopists. The endoscopists in this study have rich experience in the operation of colonoscopy. This study is a single center study, and the small number of cases studied, the results of certain limitations. In addition, due to the limitations of this study, the results might be different from those in other hospitals. For example, an average of time to arrive cecum of endoscopists in our hospital is 6 to 8 minutes, while that of other hospitals is 7 to 10 minutes.^[19–21]^ Based on the above reasons, a further multi-center comparative study is needed. Because of different learning atmosphere, operation practice and turnover rate of assistants in various hospitals, the influencing factors of assistants on endoscopists may be inconsistent. For example, it has been reported that the detection rate of polyp and adenoma of the inexperienced assistant is much lower that of the experienced assistant, and the rate of complication in the operation is much higher than that of the experienced assistant.^[22]^ However, the study had very intuitive results. Just like the learning curve of endoscopic beginners to learn colonoscopy, the learning curve of endoscopic assistants my also exist.^[23]^ However, due to the high mobility of assistants and the need for rich experience in gastroenterology, training cannot achieve the desired effect. For example, the assistant must be familiar with every step in the colonoscopy procedure, and make timely adjustment and intervention, and detect and record the physical signs of patient, and evaluate the pain, etc. All of them are operational accumulation. We hypothesize that the inexperienced assistant may lead to a decrease in polyp detection rate, a longer total operation time, and a lower rate to arrive cecum. Although it cannot be confirmed by our existing data, the assistant is not very familiar with the whole operation process and the unexpected situation at any time will increase the possibility above. For example, if the turning over and pressing abdomen are not cooperated, or the time to press abdomen is no clear, or are not familiar with polypectomy equipment, or even do not know how to operate, etc., these results still remain unchanged even if you add a second assistant (within 6 months of experience).^[24,25]^ In addition, it is not clear whether other gastrointestinal operations, such as endoscopy, endoscopic ultrasonograpy (EUS) or endoscopic retrograde cholangiopancreatography (ERCP), will have similar effects. Therefore, our findings need to be confirmed in other medical institutions.

In conclusion, the insufficient experience of assistant and the quality of screening colonoscopy may have clinical significance, such as the time to arrive cecum, pain score of patient. However, it is not significant in key indicators such as the rate to arrive cecum, polyp and adenoma detection. Therefore, abundant assistant and the experience of endoscopists are necessary for the high quality of colonoscopy. In this study, with the cooperation of the experienced endoscopists and skilled assistant, the most colonoscopies can pass through the sigmoid colon in the form of axial shortening. The satisfaction of patients with non-sedating colonoscopy reached 98.3%, and only a patient of 179 was changed to painless colonoscopy due to the inability to tolerate. Therefore, with the cooperation of the experienced endoscopists and skilled assistant, the colonoscopy could be performed under the condition of non-sedation, which greatly saves medical resources. During colonoscopy, the assistant should be subject to the quality standards of the endoscopist. The endoscopic supervisor should strictly examine the endoscopic quality data, so as to improve the quality of the colonoscopy and the colonoscopy sensitivity of patients.

## Author contributions

**Conceptualization:** Mugen Dai, Hua shi, Bin Ye.

**Data curation:** Lixia Fu, Mugen Dai, Yanmei Lan, Miaoxia Shen, Bin Ye.

**Formal analysis:** Lixia Fu, Mugen Dai, Bin Ye.

**Funding acquisition:** Lixia Fu, Bin Ye.

**Investigation:** Junwei Liu, Bin Ye.

**Methodology:** Lixia Fu, Junwei Liu, Jundi Pan, Bin Ye.

**Project administration:** Junwei Liu, Jundi Pan.

**Resources:** Junwei Liu, Xiaoduo Shao.

**Software:** Jundi Pan.

**Supervision:** Hua shi, Jundi Pan, Xiaoduo Shao.

**Validation:** Mugen Dai, Hua shi, Miaoxia Shen.

**Visualization:** Hua shi, Yanmei Lan.

**Writing – original draft:** Bin Ye.

**Writing – review & editing:** Lixia Fu.
